# Knowledge-guided multi-task learning with cascade out-of-distribution rejection for rice leaf disease diagnosis

**DOI:** 10.1371/journal.pone.0362080

**Published:** 2026-11-12

**Authors:** Xiang Lyu, Lan Bai, Yue Yu, LuMin Liu

**Affiliations:** 1 School of Information Engineering, Xinyang Agriculture and Forestry University, Xinyang, China; 2 School of Management, Xinyang Agriculture and Forestry University, Xinyang, China; Universidade Federal de Uberlandia, BRAZIL

## Abstract

Accurate diagnosis of rice leaf diseases is essential for timely crop protection, but most deep learning classifiers offer little insight into the visual evidence behind their predictions and assign a disease label even to non-target images. This study aimed to develop a diagnostic framework that integrates phytopathological knowledge into deep learning and rejects out-of-distribution (OOD) inputs. We propose the Knowledge-Guided Multi-Task Rice Network (MTRNet), which attaches a disease-diagnosis head and three auxiliary heads for lesion shape, color, and location to a shared ResNet-50 backbone. An expert knowledge matrix automatically generates the attribute labels, providing knowledge-guided supervision without pixel-level annotation. At inference, a two-stage Cascade Inference System (CIS) combines a color-based biological plausibility filter with a consistency check between the predicted disease and its predicted attributes. MTRNet was evaluated on a public dataset of 5,932 images of four rice diseases (Leaf Blast, Brown Spot, Bacterial Leaf Blight, and Tungro) and on 1,000 OOD images, using source-aware five-fold cross-validation that keeps all copies of each source photograph within a single partition. MTRNet achieved an accuracy of 96.32% ± 1.45% and a macro-F1 score of 96.43% ± 1.34%, while providing attribute-level predictions for every diagnosis. The CIS rejected 77.78% ± 0.63% of OOD images while retaining 91.71% of genuine diseased leaves, and a split-sample analysis showed that threshold selection introduced negligible optimism (about 0.2 percentage points). The framework adds only 0.027 M parameters to the backbone, and the plausibility filter requires 0.34 ms per image. These results indicate that combining knowledge-guided multi-task learning with cascade rejection enables accurate, attribute-informed, and computationally efficient rice disease diagnosis with a built-in safeguard against non-target inputs, providing a practical basis for reliable image-based disease screening in agriculture.

## 1. Introduction

### 1.1. Background and significance

As a staple food for over half of the global population, rice (Oryza sativa) plays a critical role in maintaining global food security and social stability [1]. However, throughout its growth cycle, the crop remains highly vulnerable to a wide range of pathogenic fungi, bacteria, and viruses. Common rice diseases, such as rice Leaf Blast, bacterial leaf blight, and brown spot, are characterized by high infectivity and destructive potential. According to a global assessment by Savary et al. [2], pathogens and pests cause an estimated 30.0% loss in global rice production, a figure that is particularly devastating for regions facing food shortages. Consequently, rapid and accurate early diagnosis is paramount for implementing precision agriculture and safeguarding crop yields against these biotic stresses [3]. Traditionally, disease identification has relied on visual inspections by agricultural experts. This manual process is not only labor-intensive and inefficient but also highly subjective, often leading to misdiagnosis due to visual fatigue or a lack of specialized expertise. In the early phase of precision agriculture, disease diagnosis relied primarily on handcrafted descriptors coupled with traditional classifiers. For instance, Phadikar et al. utilized rough set theory for feature selection [4], while Kusanti and Haris applied GLCM texture features [5]. Similarly, Jeyalakshmi and Radha demonstrated the feasibility of SVM-based classification in controlled settings [6]. However, these methods often struggled to generalize across the complex variability of field environments. To bridge this gap, hybrid approaches combining deep features with traditional classifiers were explored by Shrivastava et al. and Sethy et al. [7,8]. The advent of Convolutional Neural Networks (CNNs) marked a paradigm shift. In a seminal study, Mohanty et al. demonstrated that deep models could automatically learn hierarchical representations from raw pixels, significantly outperforming traditional methods [9]. Given the scarcity of annotated agricultural datasets, transfer learning has become a standard protocol. Extensive benchmarking studies, such as those by Krishnamoorthy et al. [10] and Arora et al. have established VGGNet, ResNet, and DenseNet as robust baselines for crop disease identification [11]. Furthermore, recent work by Hasan et al. optimized lightweight architectures like MobileNet for mobile deployment, successfully balancing accuracy with computational efficiency [12]. Despite these successes, standard CNNs typically treat diagnosis as a holistic classification task, often ignoring localized symptom details [13]. To achieve pixel-level precision, researchers have historically employed semantic segmentation techniques. Methods based on color thresholding, such as those by Abbas et al. and Trivedi et al., along with recent comparative studies by Kappali et al., have demonstrated the ability to isolate disease areas for severity estimation [14–16]. However, these approaches often require computationally intensive processing and expensive pixel-level annotations. Consequently, Multi-Task Learning (MTL) has emerged as a data-efficient alternative. By branching into auxiliary tasks, MTL allows models to learn robust representations without dense supervision. Li et al. proposed YOLO-FD for simultaneous detection and segmentation in aquaculture, while Chen et al. developed MTD-YOLO for tomato maturity detection [17,18]. Furthermore, Liu et al. presented a multi-task distillation learning framework, proving that disentangling features into classification and severity tasks can significantly boost performance [19]. However, most existing MTL frameworks rely on generic auxiliary tasks and rarely incorporate domain-specific phytopathological knowledge, limiting their logical consistency.

### 1.2. Problem statement

With the advent of deep learning, Convolutional Neural Networks (CNNs) have achieved remarkable success in closed-set benchmarks. However, deploying these models in complex open-field scenarios remains challenging due to two critical limitations:

**1. Lack of Phytopathological Interpretability:** Rice disease diagnosis is intrinsically a Fine-Grained Visual Categorization (FGVC) task, characterized by high inter-class similarity and significant intra-class variance. To address this, attention mechanisms have been widely integrated to force networks to focus on discriminative lesion regions, a trend reviewed by Lu et al. [20]. The integration of specialized attention blocks has marked a significant leap in performance. For instance, Zeng et al. proposed the ICAI-V4 model, which integrates coordinate attention to capture long-range dependencies in complex backgrounds [21]. Similarly, Wang et al. introduced the CA-ENet for apple diseases, utilizing coordinate information to enhance feature localization [22]. The efficacy of Squeeze-and-Excitation (SE) blocks has also been validated by Naik et al. and Ali et al., showing improved sensitivity to subtle disease features [23,24]. Recent advances also include multi-level feature fusion and self-attention mechanisms. Sunil and Jaidhar utilized adaptive channel and spatial attention for tomato disease classification [25], while Zeng and Li explored self-attention CNNs to model local lesion contexts [26]. Although these methods improve accuracy, Cheng and Li noted that they often operate as “black boxes,” lacking explicit interpretability regarding the biological attributes (e.g., shape, color) used for decision-making [27]. Most existing methods, including recent multi-task learning approaches [28], primarily focus on pixel-level segmentation or severity regression. They fail to explicitly model the intrinsic morphological attributes (e.g., lesion shape, coloration) that human experts rely upon. For instance, early-stage rice blast (spindle-shaped) and brown spot (round/oval) exhibit high visual similarity in texture. Without explicit guidance from morphological features, models struggle to distinguish between these biologically analogous diseases.

**2. Vulnerability to Out-of-Distribution (OOD) Noise:** A critical yet frequently overlooked challenge in real-world deployment is Open-Set or Out-of-Distribution (OOD) detection. Various frameworks typically assume that test data belongs strictly to predefined training categories [29,30]. Standard classifiers operate under a “closed-world assumption,” which forces any input—even non-agricultural noise such as soil or animals—to be categorized into predefined disease classes with high confidence [31]. They lack a mechanism to reject non-agricultural objects (OOD samples). Consequently, when confronted with field interference such as gray flowerpots, soil, or animals, these models tend to produce overconfident yet erroneous predictions (e.g., misclassifying a gray pot as bacterial leaf blight due to color similarities), which severely limits their practical reliability.

### 1.3. Knowledge-guided deep learning and research gap

Integrating prior knowledge into data-driven models—termed informed machine learning [32] or theory-guided data science [33]—aims to reduce the reliance on large labeled datasets and to constrain models toward domain-consistent solutions. From a learning-theoretic perspective, auxiliary tasks that share a representation with the main task act as an inductive bias: they restrict the hypothesis space to features that are useful for several related tasks, which can improve generalization when data are limited [34]. Existing knowledge-guided approaches differ mainly in where the knowledge enters the model. Attribute-based classification encodes classes as vectors of semantic attributes and has enabled recognition of unseen categories [35]; concept bottleneck models force predictions to pass through human-interpretable concepts that must be annotated for every training image [36]; and prototype-based networks explain decisions by comparing image regions with learned prototypical parts [37]. In plant disease diagnosis, prior knowledge has mostly entered indirectly, through attention modules or segmentation-based severity tasks [21,28], rather than as explicit symptom descriptors. Moreover, these knowledge-guided and interpretable models are typically evaluated under a closed-set assumption, whereas OOD detection is usually treated as a separate, confidence-based post-processing problem [31,38].

MTRNet occupies a middle ground between these lines of work. Unlike concept bottleneck models, it requires no per-image concept annotation, because attribute targets are derived from a class-level expert matrix; unlike generic multi-task learning, its auxiliary tasks are defined by phytopathological symptom descriptors; and unlike confidence-based OOD detectors, it reuses the same expert matrix at inference to check whether the predicted disease and its predicted attributes are mutually consistent. Because the attribute targets are a deterministic function of the class label, we regard this design as knowledge-guided auxiliary supervision and regularization, not as a mechanism that independently discovers disentangled pathological features.

### 1.4. Proposed solution

To bridge the gap between data-driven deep learning and domain-specific phytopathology, we propose the Knowledge-Guided Multitask Rice Network (MTRNet). Unlike standard classifiers, MTRNet is inspired by the symptom-based reasoning of plant pathologists. Leveraging a ResNet-50 based multi-head architecture, the network simultaneously predicts disease categories and their associated morphological attributes, including shape, color, and location. Specifically, the ‘guidance’ is realized through knowledge-guided auxiliary supervision: a predefined expert matrix supplies attribute-level targets that regularize the shared representation toward symptom-related cues. Furthermore, the ‘location’ attribute is identified not via traditional explicit bounding boxes, but through a weakly-supervised spatial-aware mechanism that captures the macro-distribution patterns (e.g., starting at leaf margins vs. whole leaf) inherent in the convolutional feature maps.

At the core of our approach is an Expert Knowledge Injection (EKI) module, which maps disease categories to their respective morphological criteria (e.g., Rice Leaf Blast → Spindle-shaped + Gray). This generates auxiliary supervision signals without the requirement for expensive pixel-level annotations. Furthermore, we integrate a cascaded rejection system—combining a Biological Grayscale Filter and Visual Consistency Check (VCC)—to reject far out-of-distribution inputs.

The objectives of this study were (i) to design a multi-task network in which expert symptom knowledge provides auxiliary supervision without additional annotation, (ii) to design an inference-time mechanism that rejects non-target inputs, and (iii) to quantify diagnostic accuracy, OOD rejection, false rejection, and computational cost under an evaluation protocol that controls for near-duplicate leakage.

### 1.5. Main contributions

The novelty and main contributions of this study are as follows:

1. **Knowledge-guided auxiliary supervision without per-image attribute annotation.** We translate phytopathological symptom descriptors (lesion shape, color, and onset location) into a class-level expert matrix that automatically generates attribute targets for three auxiliary heads, providing structured attribute-level outputs at a cost of only 0.027 M additional parameters.
2. **Dual use of expert knowledge for training and rejection.** The same expert matrix that supervises training is reused at inference in a two-stage Cascade Inference System, which combines a color-based plausibility filter with a disease–attribute consistency check to reject far-OOD inputs. We quantify the contribution of each stage through an ablation study.
3. **A leakage-aware evaluation protocol.** We show that the widely used public 5,932-image rice leaf dataset consists of flipped and rotated copies of approximately 321 source photographs, report source-aware cross-validation alongside image-level results, and separately assess threshold selection and evaluation bias for OOD rejection.

## 2. Methodology

### 2.1. Data materials and experimental setup

This study used two image sets: a public rice disease dataset for representation learning and an Environmental Noise Dataset (END) of non-target images for evaluating rejection.

1. Rice Disease Dataset (positive samples): This study used the publicly available Rice Leaf Disease Image Samples dataset [39], which contains 5,932 RGB images of four biotic stresses: Rice Leaf Blast (1,440 images), Brown Spot (1,600), Bacterial Leaf Blight (1,584), and Tungro (1,308). Images of Leaf Blast, Brown Spot, and Bacterial Leaf Blight are 300 × 300 pixels, whereas Tungro images have non-square, varying resolutions; all images show single leaves or leaf segments against field or simple backgrounds (Fig 1). Because class-dependent image geometry could act as a shortcut cue after resizing, we note it as a characteristic of the dataset. No acquisition metadata (plant, field, or session identifiers) are provided. A perceptual-hash analysis invariant to 90° rotations and flips (Hamming distance ≤ 8 of 64 bits, verified by pixel correlation; S1 Appendix) showed that the images form approximately 321 near-duplicate clusters (median 16 images per cluster, all class-pure), i.e., the dataset largely consists of rotated and flipped copies of a smaller set of source photographs. Consequently, a conventional image-level split places near-duplicates of every validation and test image in the training set, a form of data leakage known to inflate reported performance [40,41]. We therefore used two protocols: (i) an image-level stratified 8:1:1 split, retained for comparability with previous studies on this dataset, and (ii) a source-aware five-fold cross-validation in which each near-duplicate cluster was assigned to a single partition (class-stratified; 70% training, 10% validation, and 20% test per fold). Protocol (ii) is used for all headline diagnostic results.

**Fig 1:**
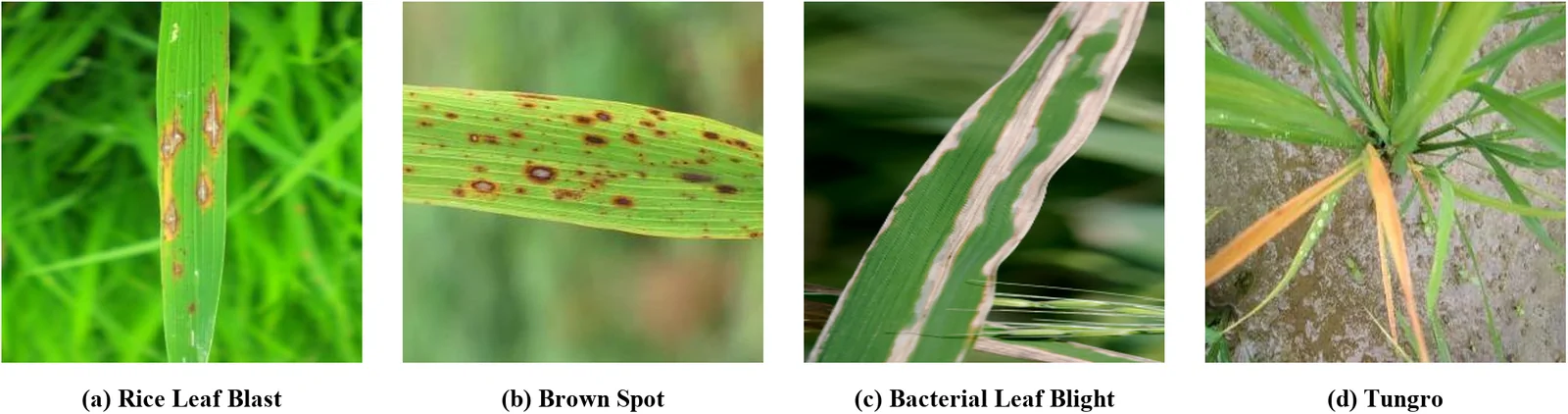
Representative samples of the four rice disease categories in the dataset. (a) Rice Leaf Blast: Characterized by spindle-shaped lesions with necrotic grey centers and brown margins. (b) Brown Spot: Typically presents as round, oval, **or narrow** dark brown spots scattered across the leaf surface. (c) Bacterial Leaf Blight: Manifests as elongated wavy lesions turning yellow or white, primarily starting from the leaf tips or margins.(d) Tungro: Exhibits diffuse chlorosis (yellow-orange discoloration) spreading from the tip.

2. Environmental Noise Dataset (negative samples): To evaluate the rejection of non-target inputs, we assembled 1,000 images of cats (497) and dogs (503). Of these, 998 were randomly sampled from the Microsoft Kaggle Cats and Dogs dataset [42] and two additional cat photographs were obtained separately. Most images show pets in domestic or urban settings; a minority show animals in outdoor or vegetated scenes (e.g., Fig 9). These images were never used for training the network. However, they were used to select the Stage I thresholds (see below), so we additionally report a split-sample analysis that separates threshold selection from evaluation. Because the set covers only two semantic categories that are visually distant from rice leaves, it represents far-OOD conditions. The detailed category distribution across these splits is illustrated in Fig 2.

**Fig 2:**
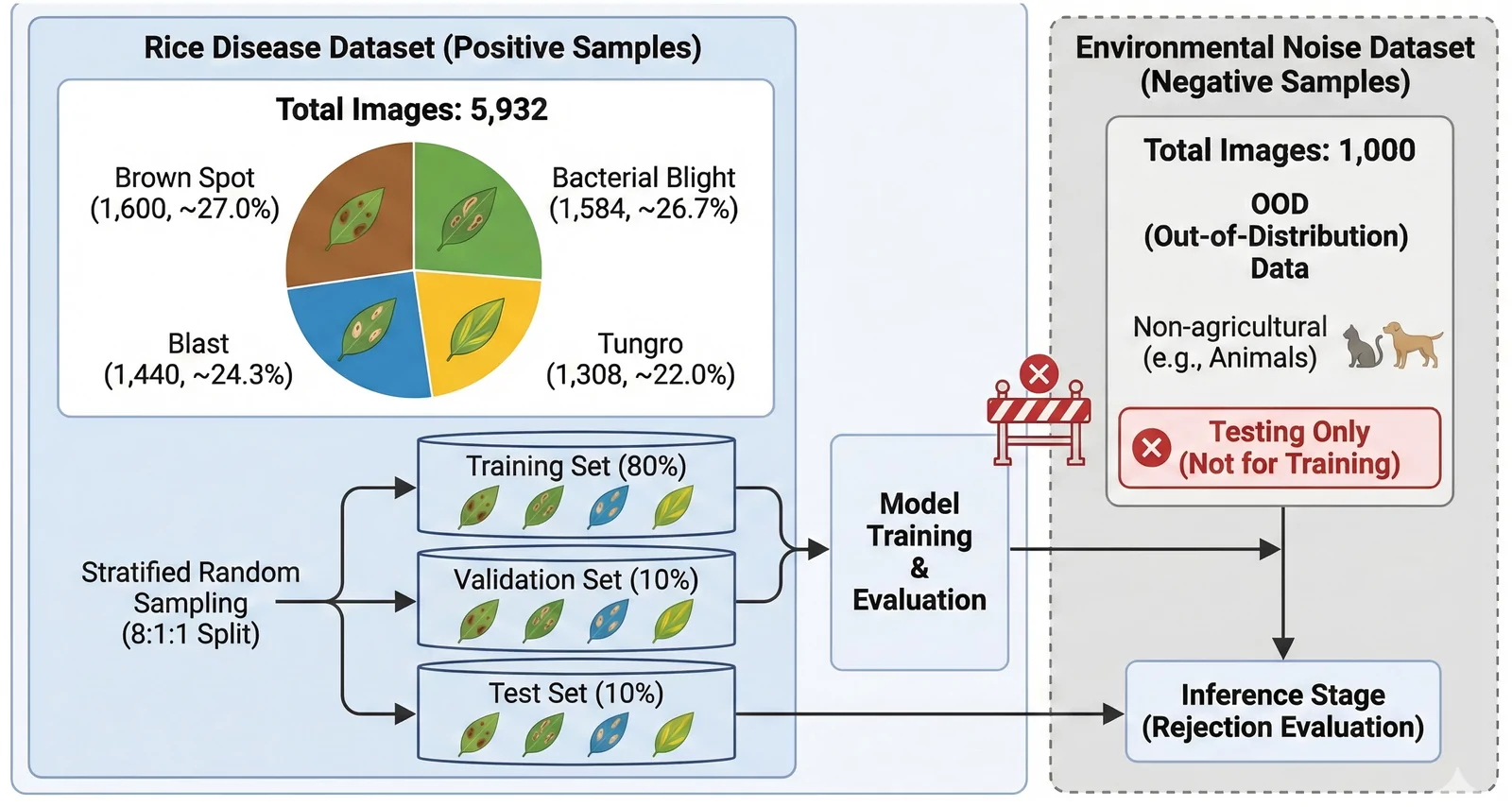
Distribution of the Dataset.

### 2.2. Overview of the framework

The proposed Knowledge-Guided Multitask Rice Network (MTRNet) is designed to combine attribute-level outputs with the rejection of out-of-distribution inputs for rice leaf disease diagnosis. As illustrated in Fig 3, the framework comprises three core components:

**Fig 3:**
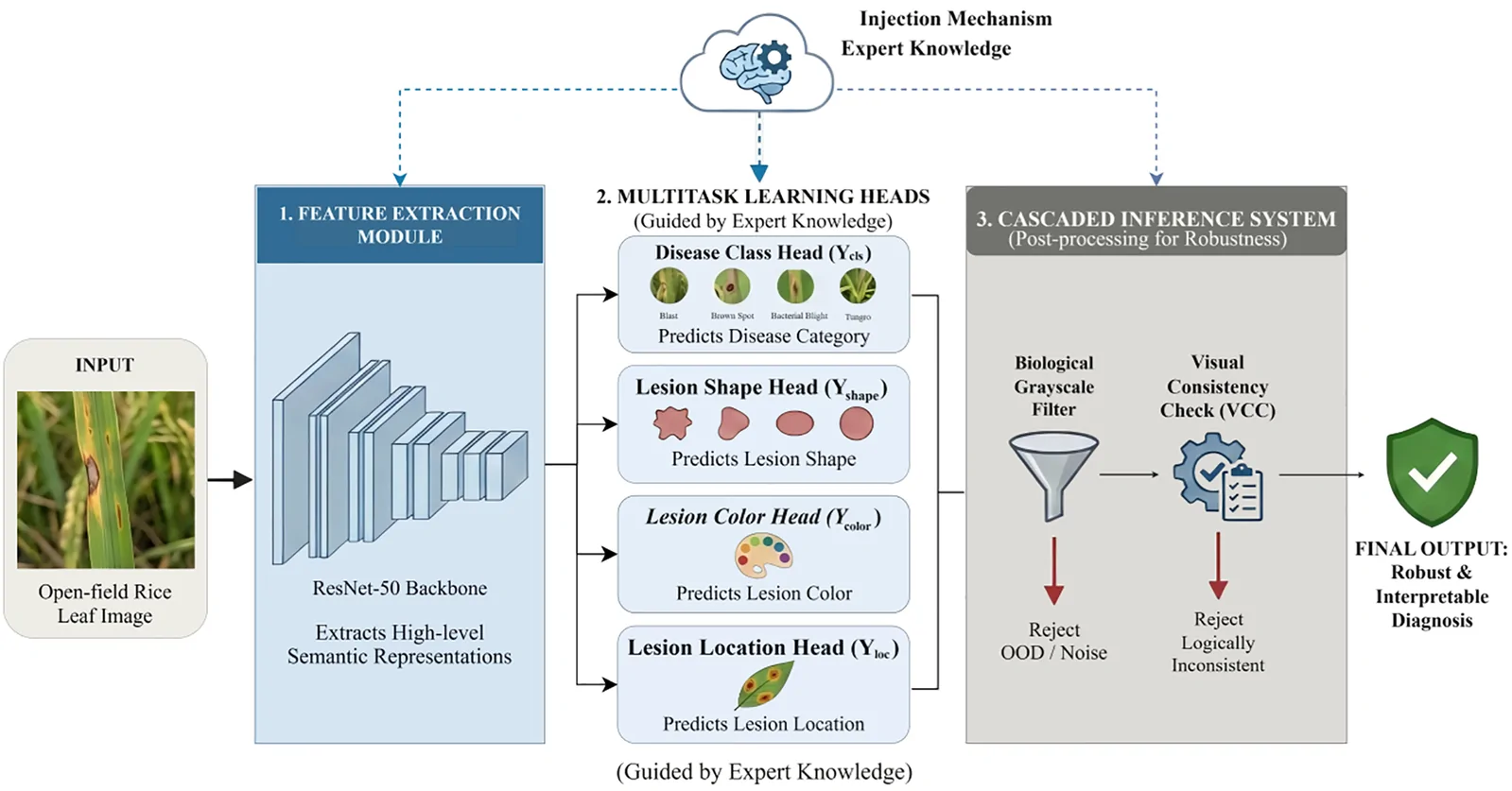
The Knowledge-guided Multitask Rice Network (MTRNet) Framework.

1. Feature Extraction Module: A ResNet-50 backbone is employed to extract high-level semantic representations from the input leaf images.
2. Multi-Task Learning Heads: Deviating from traditional single-branch classifiers, we design a multi-head architecture that simultaneously predicts the disease category (${\text{Y}}_{\text{cls}}$)—encompassing Rice Leaf Blast, Brown Spot, Bacterial Leaf Blight, and Tungro—and its three associated morphological attributes: lesion shape ($Y_{shape}$), lesion color (${\text{Y}}_{\text{color}}$), and lesion location (${\text{Y}}_{\text{loc}}$). This process is guided by an Expert Knowledge Injection (EKI) mechanism. Specifically, this mechanism maps each disease category to a predefined set of phytopathological rules (e.g., Rice Leaf Blast → Spindle-shaped + Grey/Brown). These rules automatically generate auxiliary ground-truth labels for the attribute heads, encouraging the shared representation to encode symptom-related cues without requiring expensive pixel-level annotations.
3. Cascaded Inference System: To reject non-target inputs, a post-processing module is integrated, featuring a Biological Grayscale Filter and a Visual Consistency Check (VCC). This system is dedicated to rejecting Out-of-Distribution (OOD) noise and logically inconsistent predictions.

### 2.3. Shared feature extraction

Let $X\in R^{H\times W\times \text{3}}$ denote the input image. We deploy ResNet-50 as the backbone network Φ(·). The input image is processed to generate a feature map $F_{map}$. To obtain a compact global representation, Global Average Pooling (GAP) is applied:


$$\begin{array}{c} v=GAP\left(F_{map}\right)\in R^{\text{2}\text{0}\text{4}\text{8}} \end{array}$$ (1)

where v serves as the shared latent vector encapsulating visual information required for all downstream tasks.

### 2.4. Multi-head attribute prediction

MTRNet attaches four parallel prediction heads to the shared vector v:

1. Diagnosis Head ($P_{cls}$): Determines the taxonomic category (e.g., Rice Leaf Blast).
2. Shape Head ($P_{shape}$): Identifies the morphological geometry (e.g., Spindle).
3. Color Head $(P_{color}$): Recognizes the chromatic characteristic (e.g., Grey-Brown).
4. Location Head ($P_{loc}$): Localizes the onset region (e.g., Starts at Leaf Tip/Margin vs. Whole Leaf).

The probability distribution for each task t is computed via:


$$\begin{array}{c} P_{t}=Softmax\left(W_{t}v+b_{t}\right) \end{array}$$ (2)

### 2.5. Expert knowledge injection mechanism

To address the lack of attribute annotations, we introduce a Weakly Supervised Strategy driven by expert knowledge. We formalize a Phytopathological Rules Matrix (Table 1) that maps each disease category $y_{cls}$ to its canonical attributes. Because each attribute target is a deterministic function of the class label, the auxiliary losses introduce no information beyond the label itself; their role is to shape the shared representation. Following the inductive-bias view of multi-task learning [34], requiring the backbone to support shape, color, and location predictions encourages features that also encode these symptom cues, rather than only the minimum information needed to separate four classes. We chose class-level attribute supervision instead of concept bottlenecks [36] or segmentation-based multi-task learning [28] because it needs no per-image concept or pixel annotation, and a global-pooled ResNet-50 backbone because it is a well-established, widely benchmarked baseline for crop disease recognition [10,11].

**Table 1:** Expert knowledge matrix for rule injection.

Disease	Shape (Y_shape)	Color (Y_color)	Location (Y_loc)
**Leaf Blast**	Spindle	Grey-Brown	Random/Scattered
**Brown Spot**	Round/Oval	Grey-Brown	Random/Scattered
**Bacterial Leaf Blight**	Irregular/Whole	White/Pale	Leaf Tip/Margin
**Tungro**	Irregular/Whole	Yellow-Orange	Whole Leaf

The ground-truth labels for the auxiliary tasks are automatically generated via mapping functions F. Consequently, the total multi-task objective function $L_{total}$ is formulated as:


$$\begin{array}{c} L_{total}=\lambda_{cls}L_{CE}(P_{cls},Y_{cls})+\sum_{k\in \{shape,color,loc\}}\lambda_{k}L_{CE}(P_{k},Y_{k}) \end{array}$$ (3)

In our experiments, we prioritized diagnostic accuracy while using the attribute heads as auxiliary regularizers; accordingly, the classification weight was fixed to 1.0 and the three attribute weights to 0.5. This reflects a main-task/auxiliary-task design: because the attribute labels are deterministically derived from the class label through the expert matrix, their gradients are strongly correlated with the classification gradient, so weighting them equally (1.0) would over-constrain the shared backbone, whereas a value near zero would remove the regularization benefit. A weight of 0.5 provides a balanced compromise between classification accuracy and attribute consistency. An empirical sweep over the attribute weight in {0.25, 0.5, 1.0} on the validation set of the image-level split produced identical validation accuracy and attribute consistency (100% in all three cases), confirming that performance is insensitive to this weight within this range; we therefore retained 0.5 as a balanced default.$\lambda_{cls}=\text{1}.\text{0}and\lambda_{shape}=\lambda_{color}=\lambda_{loc}=\text{0}.\text{5}.$

### 2.6. Cascade inference system for OOD robustness

To filter out the negative samples (Environmental Noise) during deployment, we propose a Cascade Inference System (CIS).

#### 2.6.1 Stage I: Bio-Grey Filter (Biological Plausibility).

We analyze the image in the HSV (Hue, Saturation, Value) color space to verify biological plausibility. We define a valid vegetation pixel mask ${\text{M}}_{\text{bio}}$ based on the empirical thresholds described below:


$$\begin{array}{c} M_{bio}(i,j)=\left\{ \begin{array}{cc} 1, & \text{if }H(i,j)\in [12,65]\wedge S(i,j)>60\wedge V(i,j)>40\\ 0, & \text{otherwise} \end{array}\right. \end{array}$$ (4)

**Parameter Optimization via Statistical Analysis:** Instead of relying on arbitrary heuristics, the hue bounds of the Bio-Grey Filter were derived from the hue distribution of the training images ($D_{train}$). We analyzed the pixel-wise Hue distribution of all vegetation regions. As shown in Fig 4, the distribution of rice tissues (including healthy leaves and disease lesions) exhibits a distinct peak in the yellow-green spectrum. To ensure robust coverage while minimizing background noise, we calculated the Cumulative Distribution Function (CDF) of the valid pixels and selected the interval covering the central 95% of the data distribution.

**Fig 4:**
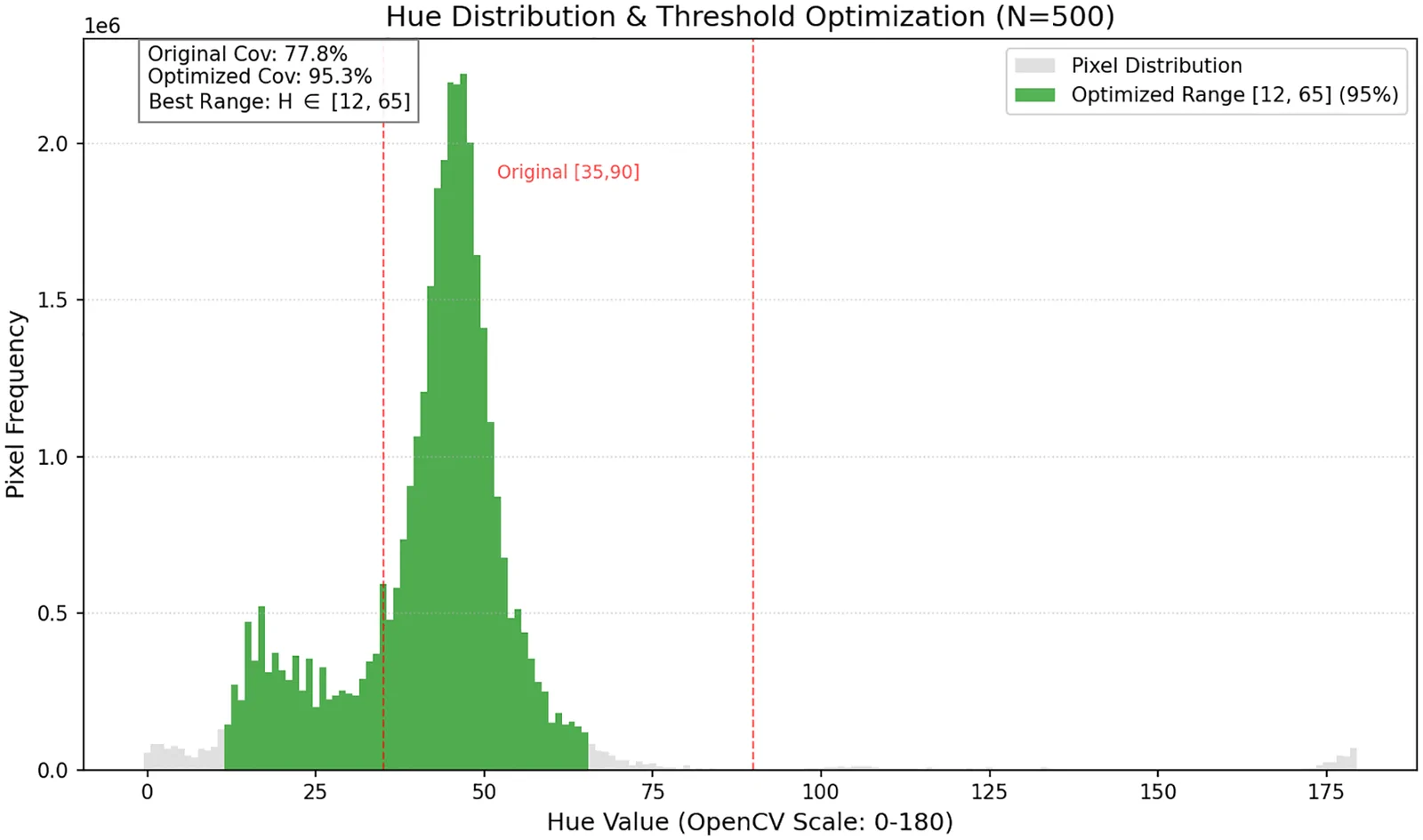
Hue Distribution & Threshold Optimization.


$$\begin{array}{c} H_{opt}=[CDF^{-\text{1}}(\text{0}.\text{0}\text{2}\text{5}),CDF^{-\text{1}}(\text{0}.\text{9}\text{7}\text{5})]\approx [\text{1}\text{2},\text{6}\text{5}] \end{array}$$ (5)

**Lower Bound (**H=12**):** Captures the chlorotic (yellowing) symptoms typical of *Tungro* and *Bacterial Blight*, effectively distinguishing them from reddish-brown soil (H<10).

**Upper Bound (**H=65**):** Covers deep green healthy tissues while filtering out cyan/blue artificial artifacts (H>70) such as sky or clothing. This data-driven strategy increased the pixel coverage rate from 88.5% (standard interval) to 95.0%, significantly reducing information loss.

The Plant Pixel Ratio (${\text{R}}_{\text{bio}}$) is calculated as $\frac{\sum {\text{M}}_{\text{bio}}}{\text{H}\times \text{W}}$. In this study, we set the strictness threshold $\tau_{\text{env}}=\text{0}.\text{35}$. If ${\text{R}}_{\text{bio}}<\text{0}.\text{35}$, the image is strictly rejected as “Non-Plant Noise,” effectively filtering out backgrounds with sparse vegetation-like artifacts.

Hyperparameter Optimization via Grid Search: Determining the thresholds for Saturation (${\text{S}}_{\text{min}}$) and Vegetation Ratio ($\tau_{\text{env}}$) involves a critical trade-off between safety (OOD rejection) and sensitivity (disease retention). To identify the optimal configuration, we conducted a grid search in which the OOD rejection rate was computed on the 1,000 Environmental Noise images and the rice retention rate on a subset of 500 rice images (404 training, 52 validation, and 44 test images of the reference split; images downscaled to a width of 320 pixels for the search). Fig 5 visualizes the sensitivity landscape.

**Fig 5:**
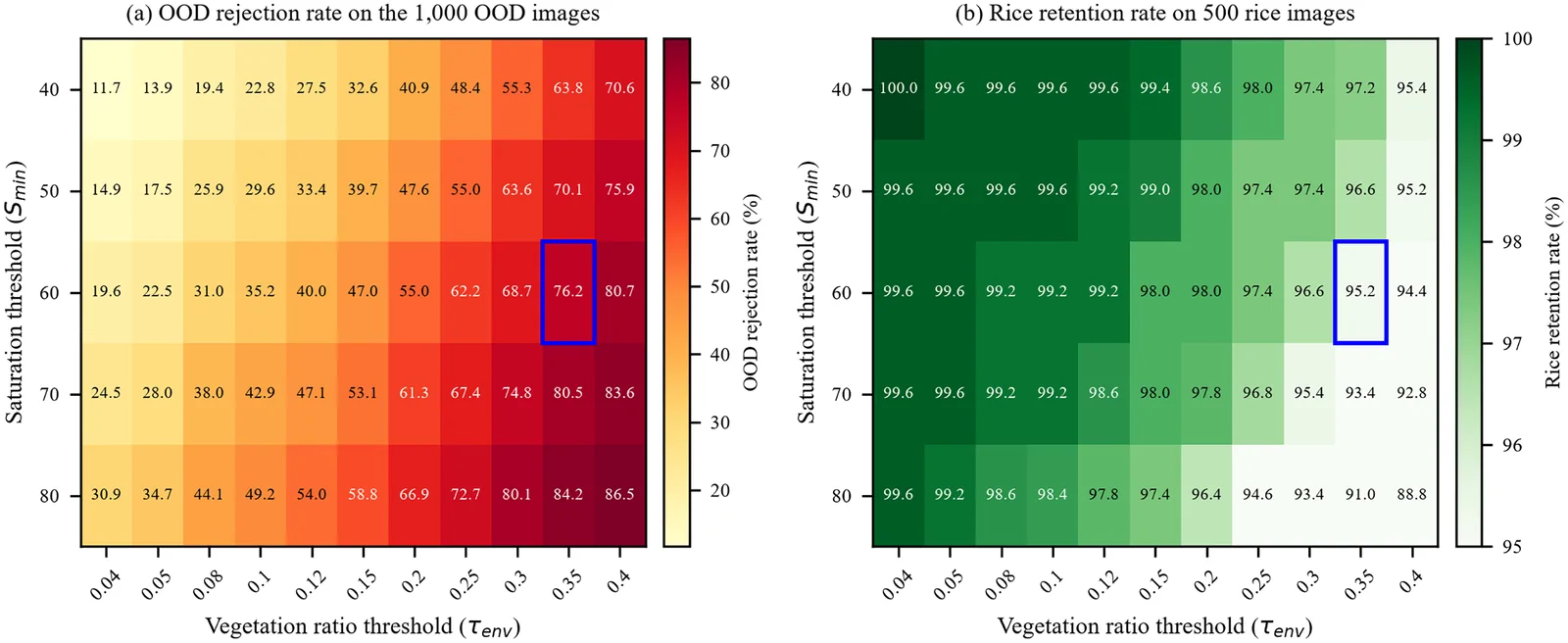
Sensitivity of the Bio-Grey Filter to the saturation threshold (S_min_) and vegetation ratio threshold (τ_env_). (a) OOD rejection rate on the 1,000 Environmental Noise images (higher is better). (b) Retention rate on 500 rice images (higher is better). The blue box marks the selected operating point (S_min_ = 60, τ_env_ = 0.35).

The OOD rejection rate (left heatmap) increases significantly as thresholds tighten. Lower thresholds (τ<0.2) fail to reject many OOD images, resulting in poor rejection performance (<40%).

Conversely, the rice retention rate (right heatmap) drops when thresholds become too strict. Notably, setting τ>0.4 leads to the accidental removal of valid samples containing small lesions, which is unacceptable for diagnostic tasks.

Selection: Based on these observations, we selected ${\text{S}}_{\text{min}}=\text{60}$ and $\tau_{\text{env}}=\text{0}.\text{35}$ as an operating point that favors OOD rejection while keeping rice retention at 95.2% on these 500 images; neighboring settings with comparable trade-offs (e.g., S_min_ = 50, τ_env_ = 0.40) yield similar results (see Robustness Evaluation on OOD Noise).

#### 2.6.2 Stage II: Visual Consistency Check (VCC).

For images passing Stage I, we perform a logical consistency check. We define a Consistency Score (CS) to verify if the predicted attributes match the diagnosis:


$$\begin{array}{c} CS=I({\hat{y}}_{shape}\in F_{shape}({\hat{y}}_{cls}))+I({\hat{y}}_{color}\in F_{color}({\hat{y}}_{cls}))+I({\hat{y}}_{loc}\in F_{loc}({\hat{y}}_{cls})) \end{array}$$ (6)

If CS<3, the prediction is flagged as “Uncertain/OOD.” This is intended to intercept images that pass the color filter but whose predicted attributes are inconsistent with the predicted disease.

The overall workflow of MTRNet, encompassing both the knowledge-guided training phase and the cascade inference phase, is summarized in Algorithm 1. This algorithmic description highlights how the expert rules are utilized for weak supervision during training and for logical consistency verification during inference.

**Algorithm 1: Training and Inference Strategy of MTRNet**

**Require:** Training set ${\text{D}}_{train}=\{(x_{i},y_{i}){\}}_{i=\text{1}}^{N}$; Test image$x_{test}$

**Ensure:** Trained model parameters $\theta^{*}$; Diagnostic result$\hat{y}$

1: Expert Knowledge Matrix M (Table 1)

2: Hyperparameters: Threshold $\tau_{env}=\text{0}.\text{35}$; Weights $\lambda_{cls}=\text{1}.\text{0}$,$\lambda_{attr}=\text{0}.\text{5}$

// Phase 1: Knowledge-Guided Training

3: Initialize model parameters θ (ResNet-50 backbone + 4 heads)

4: **for** each epoch e=1 **to** E **do**

5: **for** each batch $(x_{b},y_{b})$ in ${\text{D}}_{train}$ **do**

6: // Generate attribute labels via Expert Matrix

7: $y_{s\text{h}ape},y_{color},y_{loc}\leftarrow \text{Lookup}(y_{cls},\text{M})$

8: // Forward propagation

9: $p_{cls},p_{s\text{h}ape},p_{color},p_{loc}\leftarrow \text{MTRNet}(x_{b};\theta )$

10:// Compute multi-task loss

11: ${\text{L}}_{cls}\leftarrow \text{CrossEntropy}(p_{cls},y_{cls})$

12: ${\text{L}}_{attr}\leftarrow \sum k\in \{s\text{h}ape,color,loc\}\;\text{CrossEntropy}(p_{k},y_{k})$

13: ${\text{L}}_{total}\leftarrow \lambda_{cls}{\text{L}}_{cls}+\lambda_{attr}{\text{L}}_{attr}$

14: // Update parameters

15: $\theta \leftarrow \theta -\eta \;\nabla_{\theta }{\text{L}}_{total}$

16: **end for**

17: **end for**

18: **return**$\theta^{*}$

// Phase 2: Cascade Inference (OOD Rejection)

19: **Function**$\text{Inference}(x_{test},\theta^{*})$

20: // Stage I: Bio-Grey Filter (HSV Check)

21: Compute HSV mask $M_{bio}$ where H∈[12,65], S>60,V>40

22: Compute plant ratio$R_{bio}\leftarrow \text{Count}(M_{bio})/(H\times W)$

23: **if** $R_{bio}<\tau_{env}$ **then**

24: **return** “Non-Plant Noise (Rejected)”

25: **end if**

26: // Stage II: Visual Consistency Check (VCC)

27: ${\hat{y}}_{cls},{\hat{y}}_{s\text{h}ape},{\hat{y}}_{color},{\hat{y}}_{loc}\leftarrow \text{ArgMax}(\text{MTRNet}(x_{test};\theta^{*}))$

28: Compute consistency score:

29: $CS\leftarrow \text{1}({\hat{y}}_{s\text{h}ape}\in \text{M}[{\hat{y}}_{cls}])+\text{1}({\hat{y}}_{color}\in \text{M}[{\hat{y}}_{cls}])+\text{1}({\hat{y}}_{loc}\in \text{M}[{\hat{y}}_{cls}])$

30: **if** CS<3 **then**

31: **return** “Uncertain/ OOD (Rejected)”

32: **else**

33: **return** ${\hat{y}}_{cls}$ ▷ Valid Diagnosis

34: **end if**

35: **End Function**

### 2.7. Evaluation metrics

To comprehensively evaluate the diagnostic performance and field robustness of MTRNet, we employ two sets of metrics.

First, for the primary disease diagnosis task, we utilize standard classification metrics: Accuracy (Acc), Precision (Pre), Recall (Rec), and F1-score.


$$\begin{array}{c} Accuracy=\frac{TP+TN}{TP+TN+FP+FN} \end{array}$$ (7)

$$\begin{array}{c} F\text{1}=\text{2}\times \frac{Precision\times Recall}{Precision+Recall} \end{array}$$ (8)

Second, to quantify the model’s reliability against open-world noise, we define the OOD Rejection Rate (ORR). It represents the percentage of negative samples successfully flagged as “Non-Plant” or “Uncertain” by our Cascade Inference System (CIS):


$$\begin{array}{c} ORR=\frac{N_{rejected}}{N_{total\_negative}}\times \text{1}\text{0}\text{0}\% \end{array}$$ (9)

where $N_{rejected}$ denotes the number of samples intercepted by either the Bio-Grey Filter or the Visual Consistency Check.

In addition, to characterize the safety–sensitivity trade-off of the CIS, we report the False Rejection Rate (FRR): the proportion of genuine diseased leaves in the positive test set that are erroneously rejected by the cascade, i.e., FRR = N_falsely-rejected/ N_positive.

Two additional analyses were performed. First, to separate threshold selection from evaluation, the 1,000 OOD images were randomly split 100 times into equal tuning and held-out halves; on each tuning half, (S_min_, τ_env_) were re-selected from the grid by maximizing OOD rejection subject to the validation retention of the original operating point, and the ORR of the full CIS was computed on the held-out half. Second, computational cost was measured as parameter count, multiply–accumulate operations (MACs) at 224 × 224 input, and single-image latency on CPU (Intel Core i5-13500H, PyTorch 2.13) and GPU (NVIDIA T4).

## 3. Results

### 3.1. Performance on rice disease diagnosis

#### 3.1.1 Training convergence analysis.

On the reference image-level split, MTRNet was trained for 50 epochs. As illustrated in Fig 6, the model exhibited rapid and stable convergence.

**Fig 6:**
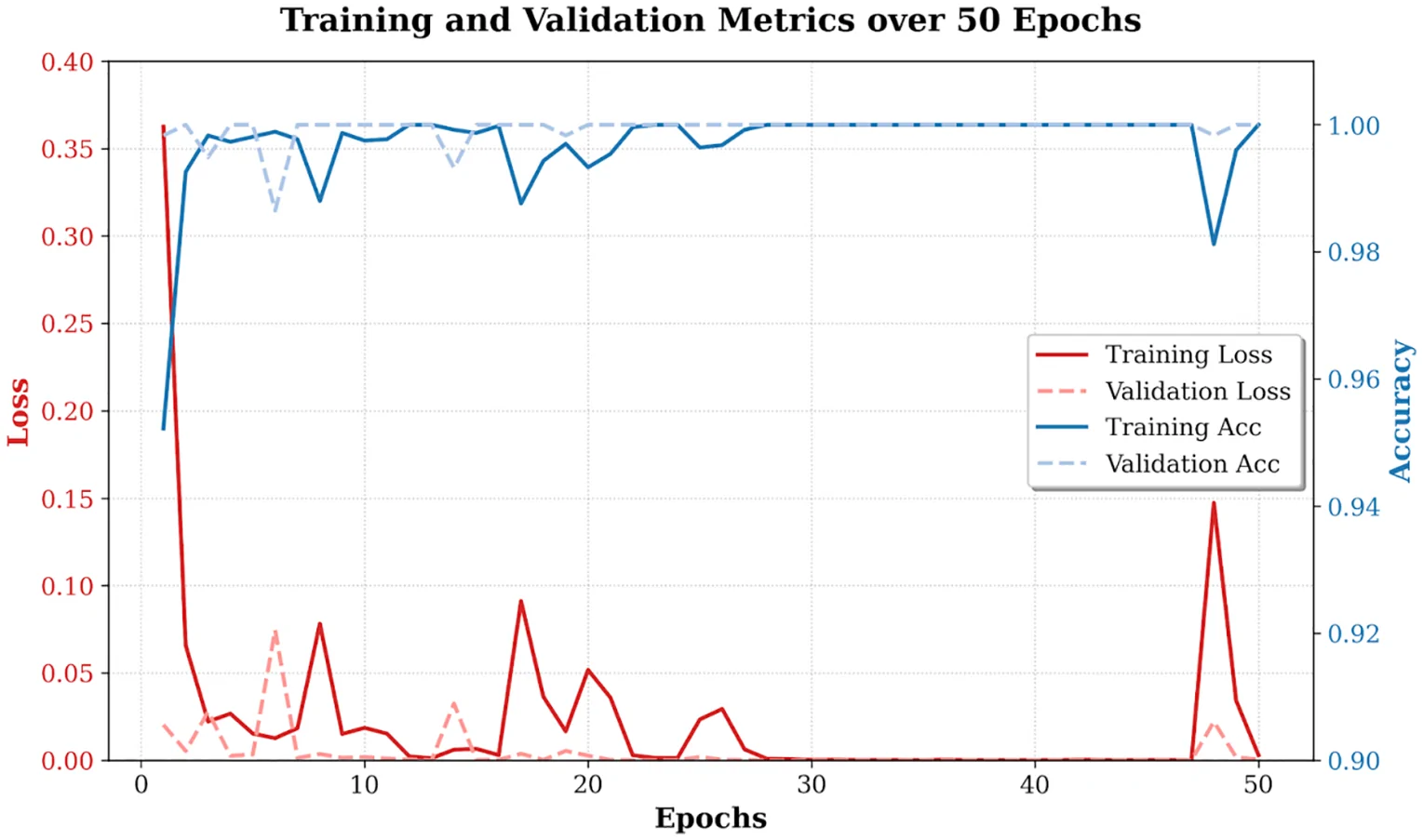
Training and Validation Metrics over 50 Epochs.

**Loss Curve:** The total multi-task loss decreased sharply within the first 15 epochs and stabilized around epoch 35, indicating stable optimization.

**Accuracy Curve: The validation accuracy steadily improved, reaching a peak of 100% at Epoch 50. We interpret this result cautiously: a high validation accuracy does not by itself establish the absence of overfitting, and it may partly reflect the relatively constrained visual difficulty of this four-class benchmark** and the presence of near-duplicate images across splits. Because image-level splits place near-duplicates of every validation image in the training set (see Data Materials), these curves reflect optimization stability only and should not be read as evidence of generalization.

#### 3.1.2 Test set evaluation.

We evaluated the best-performing model checkpoint on the reference image-level test set (595 images). MTRNet achieved an overall accuracy of 99.83%, misclassifying only 1 out of 595 samples. Fig 7 presents the Confusion Matrix. The model achieved 100% accuracy on Brown Spot, Bacterial Leaf Blight, and Tungro; the single misclassification occurred for a Rice Leaf Blast sample, which is understandable given the visual similarity between early-stage Leaf Blast and Brown Spot lesions. Because every test image has a rotated or flipped near-duplicate in the training set under this image-level split, this figure substantially overestimates performance on unseen photographs; leakage-controlled results are reported in the Source-Aware Cross-Validation subsection.

**Fig 7:**
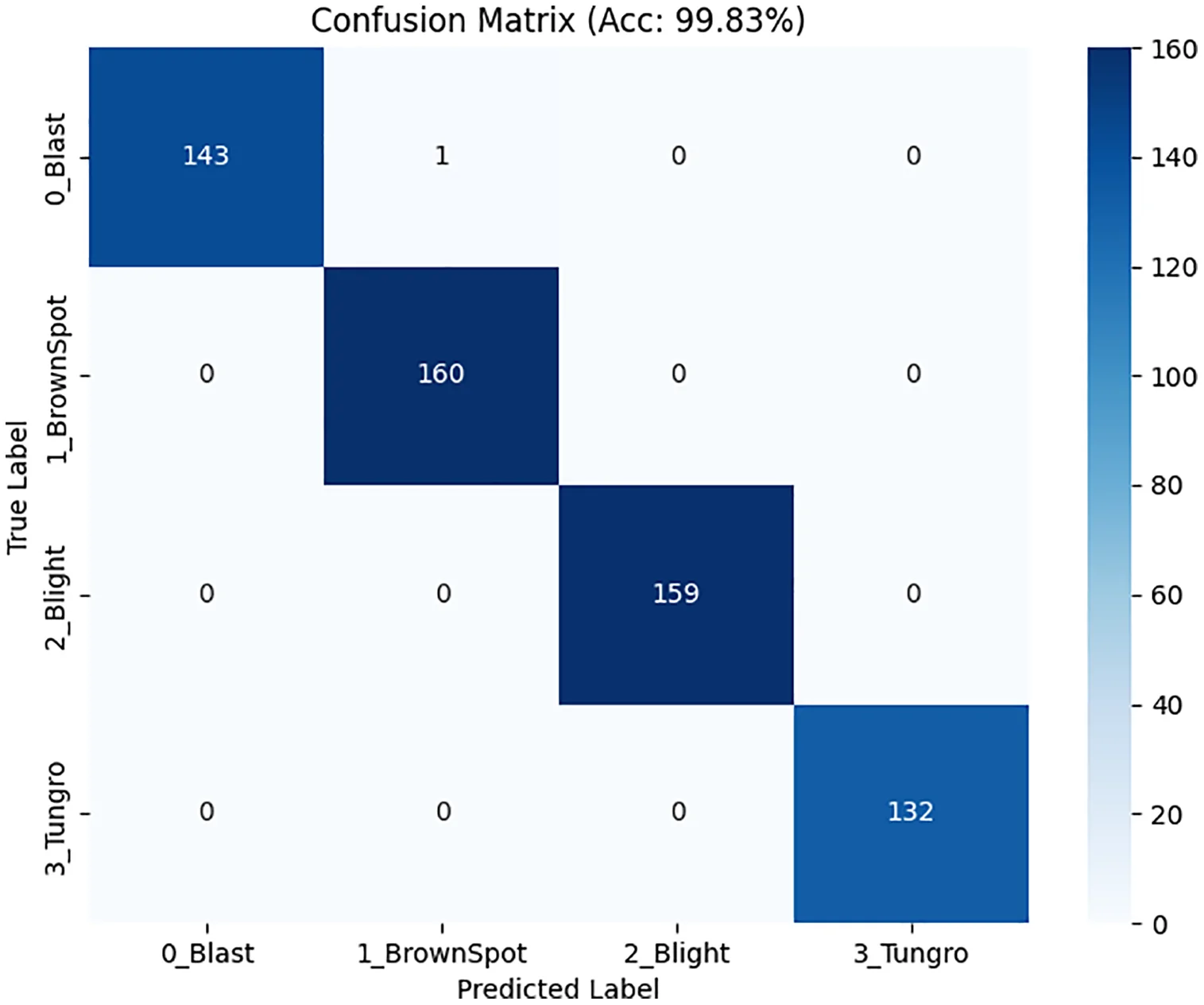
Confusion Matrix.

### 3.2. Attribute-level outputs

Beyond the class label, MTRNet outputs attribute-level predictions via its auxiliary heads. To verify this, we analyzed the output of the attribute heads. For instance, when classifying a Rice Leaf Blast sample, the network not only outputs the class label “Rice Leaf Blast” but also predicts, on average across the 144 Leaf Blast test samples:

Shape Head: Spindle (mean probability ≈ 0.99)
Color Head: Grey-Brown (mean probability ≈ 0.998)
Location Head: Random/Scattered (mean probability ≈ 0.998)

This agreement between the predicted attributes and the canonical descriptors in our Expert Knowledge Matrix shows that the auxiliary heads have learned the intended class-to-attribute mapping. We emphasize, however, that attribute–matrix consistency alone does not establish the faithfulness or causal validity of the model’s reasoning; a rigorous verification would require a blinded evaluation by plant-pathology experts with inter-rater agreement metrics, which we identify as future work. Importantly, because the attribute targets are deterministically derived from the disease label, high attribute–matrix consistency is expected by construction and does not by itself demonstrate that the network has decomposed independent pathological features. The auxiliary heads are therefore best understood as knowledge-guided attribute-level supervision that regularizes the shared representation, and their outputs as a structured restatement of the diagnosis rather than independent evidence. This is borne out by the source-aware experiments: 99.33% ± 0.28% of test images received attribute predictions fully consistent with their predicted class even though classification accuracy was 96.32%, and 197 of the 218 misclassified test images (90.4%) received attributes fully consistent with the wrong class. The attribute heads therefore largely re-encode the class decision, and the consistency check cannot detect in-distribution misclassifications.

To qualitatively examine where the attribute branches attend, we computed Grad-CAM [43] activation maps of the Location branch (Fig 8). For tip/margin-onset diseases such as Bacterial Leaf Blight, the activation concentrates on the elongated lesion along the leaf, whereas for whole-leaf diseases such as Tungro the response is diffusely distributed across the canopy—consistent with the macro-distribution patterns encoded in the Expert Knowledge Matrix. Grad-CAM provides qualitative evidence of attention localization but does not establish independent pathological feature decomposition.

**Fig 8:**
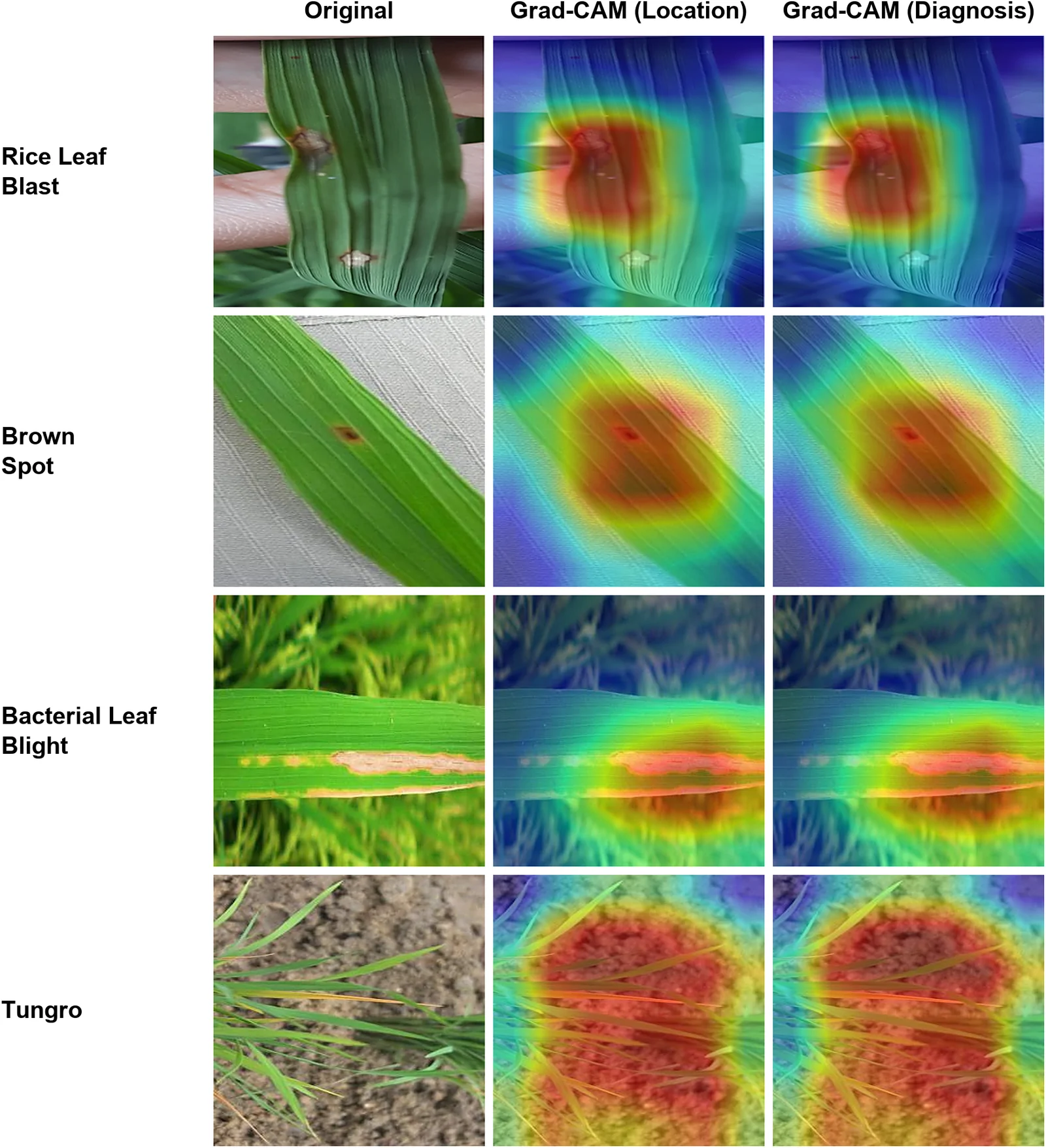
Grad-CAM verification of the Location branch. For each disease, columns show the original leaf, the Location-head activation, and the Diagnosis-head activation. Tip/margin-onset diseases (e.g., Bacterial Leaf Blight) elicit activation along the leaf margin, whereas whole-leaf diseases (e.g., Tungro) produce diffuse responses—qualitatively consistent with the onset patterns in Table 1.

### 3.3. Robustness evaluation on OOD noise

We utilized the 1,000 negative samples (Environmental Noise Dataset) to evaluate the Cascade Inference System (CIS). Using the grid search described in Methods, we set the Bio-Grey Filter parameters to H∈[12,65],S>60, and the strict vegetation ratio threshold $\tau_{env}=\text{0}.\text{3}\text{5}$. To illustrate how the two stages operate, we examined “Animal Intrusion” scenarios, which represent challenging negative examples. As shown in Fig 9, these images show dogs and cats in or near vegetated backgrounds.

**Fig 9:**
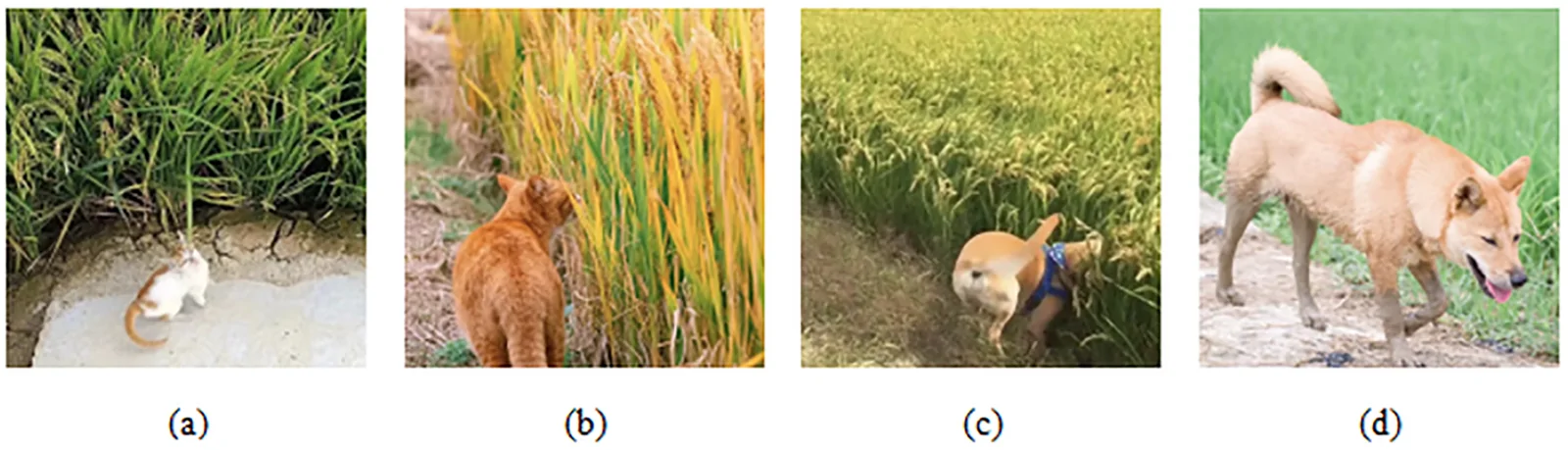
Visualization of OOD rejection results on “Hard Negative” samples (Animal Intrusion).

Rejection via Bio-Grey Filter (Stage I): For samples like Fig 9(a) and (d), the presence of barren soil and concrete significantly reduces the global vegetation ratio. Our optimized threshold ($\tau_{\text{env}}=\text{0}.\text{35}$) successfully identifies the insufficient plant pixel density, filtering out these images before they reach the deep network.

Rejection via Visual Consistency Check (Stage II): A more complex challenge is presented in Fig 9(b) and (c), where the animals are situated deeply within the dense canopy of the rice field. These images exhibit a high vegetation ratio (${\text{R}}_{\text{bio}}>\tau_{\text{env}}$) and therefore pass Stage I. The multi-task heads extract attribute probabilities (e.g., Shape: Irregular, Location: Random) that fail to match any valid row in the Expert Knowledge Matrix. Consequently, the Consistency Score drops below the threshold (CS<3), and the system correctly flags them as “Uncertain/OOD” rather than forcing a false positive diagnosis like “Leaf Blast”.

These examples are characterized by a complex mix of background vegetation (rice paddies) and non-target subjects (wandering animals). (a, d) Samples successfully intercepted by Stage I (Bio-Grey Filter). The substantial presence of concrete surfaces and muddy dirt paths significantly lowers the overall vegetation ratio, triggering an early rejection. (b, c) Samples rejected by Stage II (Visual Consistency Check). Although the dense canopy of the rice plants provides high vegetation coverage that bypasses Stage I, the predicted attributes are inconsistent with the predicted disease according to the expert knowledge matrix, so the images are rejected as out-of-distribution.

Standard Baseline: Without our rejection mechanism, a standard ResNet-50 necessarily assigns every noise image to one of the disease classes (false positive rate ≈100%).

MTRNet: Under source-aware cross-validation, the full CIS achieved a mean OOD Rejection Rate (ORR) of 77.78% ± 0.63% (95% CI [77.00%, 78.56%]); with image-level re-splits, the corresponding value was 78.16% ± 0.57%. Stage I—the model-independent Bio-Grey Filter—rejects 763 of the 1,000 negative samples (76.3%), and Stage II adds a small further gain (see Ablation of the Cascade Inference System).

We also quantified the cost of this rejection on genuine samples. Under source-aware cross-validation, the mean False Rejection Rate on the held-out positive images was 8.29% ± 2.83% (95% CI [4.77%, 11.80%]); with image-level re-splits it was 8.22% ± 1.46%. These false rejections are dominated by Stage I (7.66% ± 3.11%), where the Bio-Grey Filter conservatively rejects genuine leaves whose overall vegetation ratio is low (e.g., close-up lesion images), whereas Stage II alone rejected 0.67% ± 0.28% of genuine leaves. In practical deployment, such conservatively rejected samples would be flagged for re-imaging rather than misdiagnosed.

#### 3.3.1 Ablation of the cascade inference system.

Table 2 isolates the contribution of each stage. With the reference image-level model, Stage I alone rejected 763 of the 1,000 OOD images (76.3%) at an FRR of 6.39%, whereas Stage II alone rejected 204 (20.4%) without rejecting any genuine test leaf. Of the 237 OOD images that passed Stage I, Stage II rejected 50 (21.1%), raising the ORR of the full cascade to 81.3% with no increase in FRR. Under source-aware cross-validation, Stage I alone rejected 76.30% of OOD images at an FRR of 7.66% ± 3.11%; Stage II alone rejected 7.36% ± 2.01% at an FRR of 0.67% ± 0.28%; and the full cascade reached an ORR of 77.78% ± 0.63% at an FRR of 8.29% ± 2.83%. On average, Stage II rejected 14.8 of the 237 OOD images that passed Stage I (6.2%). Thus, most OOD rejection is achieved by the model-independent color prior of Stage I, which encodes domain knowledge about plant tissue coloration but involves no learning; the knowledge-guided consistency check of Stage II provides a small complementary gain (+1.5 percentage points in ORR under source-aware cross-validation) at a small false-rejection cost (+0.6 percentage points). The larger Stage II contribution observed with the reference image-level model (+5.0 points) did not carry over to models trained without near-duplicate leakage, so we do not regard Stage II as a major source of OOD robustness.

**Table 2:** Ablation of the Cascade Inference System (ORR and FRR, %).

Configuration	ORR, image-level model	FRR, image-level model	ORR, source-aware CV	FRR, source-aware CV
Stage I only (Bio-Grey Filter)	76.30	6.39	76.30 ± 0.00	7.66 ± 3.11
Stage II only (VCC)	20.40	0.00	7.36 ± 2.01	0.67 ± 0.28
Full CIS (Stage I + II)	81.30	6.39	77.78 ± 0.63	8.29 ± 2.83

Image-level model: the released checkpoint evaluated on the reference split (595 test images). Source-aware CV: mean ± SD over five folds. All ORR values are computed on the 1,000 OOD images.

**Threshold selection bias.** Because the Stage I thresholds were chosen using the same 1,000 OOD images, we repeated the selection on random tuning halves and evaluated the full CIS on the held-out halves (100 repetitions). The mean held-out ORR was 81.04% ± 1.39% (2.5th–97.5th percentile, 78.59–83.91%), compared with 81.22% on the tuning halves, indicating an optimistic bias of about 0.2 percentage points. The re-selected operating point was (60, 0.35) in 43 of 100 repetitions and one of two neighboring settings, (50, 0.40) or (40, 0.45), in the remainder, suggesting a flat optimum. Nevertheless, because all OOD images come from a single curated collection, the ORR should be regarded as an estimate for far-OOD inputs of a similar kind.

### 3.4 Comparison with Reported Results of Related Methods

Table 3 summarizes accuracies reported in the original publications of representative related methods, ICAI-V4 [21], PDDNet [44], and ALMDR [28]. These values were obtained on different datasets, class sets, and evaluation protocols and are therefore not directly comparable with our results; the table is intended to position MTRNet by paradigm and capability rather than to rank methods quantitatively.

**Table 3:** Reported results of related methods. Values were obtained on different datasets and protocols and are not directly comparable.

Method	Paradigm	Accuracy (%)	Explanatory output	OOD Robustness (ORR)
**ICAI-V4**	Attention	95.57%	Class label only	Not evaluated
**PDDNet**	Ensemble	97.79%	Class label only	Not evaluated
**ALMDR**	Multi-Task (Seg)	93.74%	Lesion segmentation	Not evaluated
**MTRNet**	Multi-Task (Attr)	96.32 ± 1.45 (99.83)	Class label + attribute predictions	Yes (77.78% ± 0.63%)

Values for other methods are taken from the original publications and were obtained on different datasets and protocols; they are not directly comparable. MTRNet accuracy is reported under source-aware cross-validation, with the image-level split result in parentheses.

**Annotation costP:** Distinct from approaches like ALMDR [28] that incorporate pixel-level segmentation, MTRNet adopts a weakly supervised strategy guided by expert rules. This design aims to reduce the reliance on dense annotations and computational overhead.

**Robustness:** While previous studies [19,44] predominantly operate under a Closed-World Assumption, this work explicitly addresses the rejection of non-target inputs. The proposed system achieves a mean OOD rejection rate of 77.78% ± 0.63% on far-OOD images under source-aware cross-validation.

### 3.5 Source-aware cross-validation and statistical robustness

Because image-level splits leak near-duplicates (see Data Materials), we evaluated MTRNet with source-aware five-fold cross-validation, in which no near-duplicate cluster is shared between training, validation, and test partitions. Each of the 5,932 images is held out exactly once across the folds, and hyper-parameters and model selection were identical to the original protocol. Results are summarized in Table 4. Accuracy was 96.32% ± 1.45% (95% CI 94.52–98.13%), macro-F1 96.43% ± 1.34% (95% CI 94.76–98.10%), ORR 77.78% ± 0.63% (95% CI 77.00–78.56%), and FRR 8.29% ± 2.83% (95% CI 4.77–11.80%). Accuracy was therefore about 3.5 percentage points lower than under image-level splitting, and varied more across folds (94.35–97.82%). The pooled confusion matrix over all 5,932 held-out predictions (S1 Appendix) shows that errors concentrated among the three lesion-type diseases: Bacterial Leaf Blight misclassified as Brown Spot (66 images), Leaf Blast as Bacterial Leaf Blight (63), and Leaf Blast as Brown Spot (37), whereas Tungro was recognized almost perfectly (recall 99.46%, precision 100%). Per-class recall was 93.06% (Leaf Blast), 98.62% (Brown Spot), 94.38% (Bacterial Leaf Blight), and 99.46% (Tungro).

**Table 4:** Performance under image-level re-splits (five seeds) and source-aware cross-validation (five folds). Values are mean ± SD (%) with Student-t 95% confidence intervals in brackets.

Metric	Image-level re-splits (5 seeds)	Source-aware CV (5 folds)
Accuracy	100.00 ± 0.00 [100.00, 100.00]	96.32 ± 1.45 [94.52, 98.13]
Macro-F1	100.00 ± 0.00 [100.00, 100.00]	96.43 ± 1.34 [94.76, 98.10]
OOD Rejection Rate (ORR)	78.16 ± 0.57 [77.45, 78.87]	77.78 ± 0.63 [77.00, 78.56]
False Rejection Rate (FRR)	8.22 ± 1.46 [6.40, 10.03]	8.29 ± 2.83 [4.77, 11.80]

Under image-level re-splitting, accuracy and macro-F1 were 100.00% ± 0.00% across five seeds. Because every test image has a near-duplicate in the training set under this protocol, this stability reflects consistency across random partitions of the same pool of source photographs, not generalization to new plants, fields, or acquisition sessions; the source-aware results above should be used for that purpose. The single reference split reported above yielded 99.83% accuracy (one misclassification).

For comparison with the original protocol, we also repeated the entire training and evaluation pipeline under five independent random seeds (0–4). For each seed, all 5,932 images were pooled and re-partitioned into training/validation/test sets (8:1:1) by stratified sampling, and the model was retrained from the same ImageNet-initialized backbone under identical hyper-parameters. Performance is summarized in Table 4 as Mean ± SD with 95% confidence intervals.

### 3.6 Computational cost

MTRNet adds four linear heads (0.027 M parameters) to a ResNet-50 feature extractor, giving 23.53 M parameters and 4.09 G MACs for a 224 × 224 input (90 MB checkpoint). On a laptop CPU (Intel Core i5-13500H, PyTorch 2.13), single-image network inference took 77 ms with 16 threads and 172 ms with a single thread, and 5.8 ms on an NVIDIA T4 GPU. Stage I requires 0.34 ms per image, and Stage II only compares three predicted attribute indices with a table lookup, so the CIS adds negligible overhead to a standard ResNet-50 classifier. Training for 50 epochs took approximately 44 min per fold on a T4 GPU.

## 4. Discussion

### 4.1 Knowledge-guided attribute supervision and its interpretive limits

The primary challenge in applying deep learning to precision agriculture is the inherent “black-box” nature of conventional convolutional neural networks. While existing models achieve high classification accuracy on closed-set benchmarks, their decision-making processes remain opaque to agronomists. The proposed MTRNet, built upon a ResNet-50 backbone, takes a step toward addressing this by moving from a purely data-driven paradigm to a knowledge-guided framework.

By replacing the standard fully connected layer with a diagnosis head and three attribute heads supervised through the expert matrix, MTRNet produces attribute-level outputs (shape, color, and location) alongside each diagnosis without requiring pixel-level annotation. We emphasize that these outputs should not be interpreted as evidence of explicit feature disentanglement: because the attribute targets are a deterministic function of the class label, a network can achieve high attribute consistency simply by re-encoding its class decision. The design is therefore more accurately described as knowledge-guided attribute-level auxiliary supervision that regularizes the shared representation. The Grad-CAM maps in Fig 8 qualitatively suggest that the Location branch responds to spatially plausible regions, but they do not demonstrate independent pathological feature decomposition. Demonstrating genuine disentanglement would require attribute annotations that vary independently of the class label (e.g., atypical lesion presentations), which the current dataset does not provide.

While MTRNet provides structured attribute-level outputs through its auxiliary heads, its explanation mechanism remains at the level of categorical post-hoc verification—it outputs what attributes are predicted (e.g., “Spindle-shaped”) but does not explain why specific visual regions support this prediction. This contrasts with recent advances in post-hoc interpretability. For instance, Huang et al. [45] employed an XGBoost–SHAP framework to extract scale-sensitive, non-linear variable–response relationships in ecological systems, enabling both global feature importance and local, sample-level explanations through Shapley-value-based additive decomposition. Integrating such granular attribution—such as SHAP values for each attribute head or gradient-based activation maps (as illustrated by our Grad-CAM analysis)—into MTRNet could further bridge the gap between structural transparency and mechanistic understanding, particularly for identifying which lesion regions drive specific phytopathological predictions.

### 4.2 Out-of-distribution rejection in agricultural deployment

Moving beyond standardized laboratory datasets, real-world deployment faces severe challenges from open-world environmental noise. In non-standardized agricultural environments, such as smallholder farms, data acquisition via automated UAVs or IoT cameras frequently captures unconstrained macro-level anomalies, including non-target crops (e.g., tomatoes), field weeds, or even wandering village animals. Traditional single-task classifiers often force these anomalous inputs into one of the predefined disease categories, resulting in false positives.

The integration of the Cascade Inference System (CIS) adds a rejection option for far-OOD inputs. By sequentially applying a biological grayscale filter and a visual consistency check, the system screens inputs before a diagnosis is issued. If the extracted visual features contradict the predefined phytopathological expert matrix (e.g., animal fur textures falsely triggering the “Spindle” shape attribute), the CIS rejects the sample as Out-of-Distribution (OOD) noise. In our experiments, however, most rejections were produced by Stage I (see Ablation of the Cascade Inference System), so this mechanism mainly reduces the risk that clearly non-target images receive a disease label.

It is important to delineate the operational boundary of the current robustness evaluation. The 1,000 negative samples used here are cat and dog photographs, i.e., far-OOD instances that are visually divergent from crop canopies. In real deployments, the more demanding threats are near-OOD anomalies—non-target companion weeds, unrecorded minor diseases, or mechanical leaf tears that mimic true lesions—whose low-level color and texture statistics overlap substantially with genuine rice tissue. Because Stage I operates on color plausibility and Stage II on attribute–matrix logic, near-OOD weeds that share the vegetation color profile can bypass Stage I, so their rejection depends entirely on the consistency check. We therefore regard the reported ORR as an estimate under far-OOD conditions and identify systematic near-OOD benchmarking as a necessary direction for future validation.

### 4.3 Limitations and system positioning

Despite achieving high diagnostic accuracy within the verified benchmark, a critical limitation of current RGB-based diagnostic frameworks, including MTRNet, is the inherent difficulty in distinguishing specific viral infections from abiotic stresses. For instance, the macroscopic symptoms of Tungro disease share a high visual similarity with systemic nutrient deficiencies (e.g., nitrogen or zinc deficiency), both primarily manifesting as leaf chlorosis.

While the multi-task constraint of MTRNet (such as the spatial requirement for symptoms to start from specific leaf regions) is intended to reduce false positives, we acknowledge that relying solely on single RGB images cannot definitively rule out nutritional disorders in complex field conditions. Therefore, in practical agricultural pipelines, MTRNet is best positioned as a visual triage system. A prediction of “Tungro” serves as a morphological alert based on visual probability, prompting farmers or automated systems to conduct necessary secondary agronomic verifications.

A further limitation stems from the static, manually defined Phytopathological Rules Matrix (Table 1). Under unconstrained field conditions this introduces rigidity: compound co-infections and chronological symptom transitions—where early-stage lesions deviate from the canonical descriptors—are not represented by any single matrix row. Enforcing a hard consistency requirement (CS = 3) under such variation can raise the false-rejection rate, as atypical yet genuine cases fail the logic check; this is consistent with the Stage-I-dominated false rejections reported above. The present matrix therefore best characterizes typical single-infection presentations, and future work could relax the hard threshold toward a soft, probability-weighted consistency score or learn stage-conditioned attribute rules to accommodate disease progression.

**Data independence.** The benchmark consists of flipped and rotated copies of approximately 321 source photographs, and no plant-, field-, or session-level metadata are available. Our source-aware protocol removes near-duplicates detected by a rotation- and flip-invariant perceptual hash, but it cannot exclude correlations between distinct photographs of the same plant, field, or acquisition session, or near-duplicates produced by transformations the hash does not capture (e.g., cropping or color changes). The source-aware results should therefore be regarded as an upper bound on performance for images collected independently, and external validation on field images acquired under different conditions is required before deployment.

**Consistency check.** Because attribute predictions largely follow the predicted class (90.4% of misclassified images passed the consistency check), Stage II offers little protection against confusions among the four known diseases and contributes only modestly to OOD rejection.

**Scope of OOD evaluation.** The 1,000 OOD images consist only of cat and dog photographs and are therefore far-OOD, and the Stage I thresholds were selected on this collection; although the split-sample analysis indicated little selection bias, the reported ORR does not characterize near-OOD inputs such as weeds, other crops, unseen diseases, or abiotic stress.

**Computational limitations and scalability.** Although the CIS adds negligible overhead, the ResNet-50 backbone (23.5 M parameters, 4.09 G MACs, 77–172 ms per image on CPU) is relatively heavy for low-power edge devices, and processing high-resolution UAV imagery would require tiling or detection front-ends, which multiply the inference cost. Scaling to more diseases is also constrained by the expert matrix: each new class requires expert-defined attribute rules, retraining of all heads, and possibly new attribute vocabularies, and diseases with overlapping symptoms may not be separable by three categorical attributes. Future work will investigate lightweight backbones (e.g., MobileNet- or EfficientNet-class models [12]), quantization and pruning for on-device inference, batched or streaming processing for UAV and IoT pipelines, and semi-automatic construction of attribute rules from plant pathology literature.

### 4.4 Future work

Future research will focus on addressing these limitations by incorporating multi-modal data streams. Fusing standard RGB imagery with multispectral data or real-time soil sensor metrics could provide the physiological context needed to definitively separate viral infections from abiotic stresses. Additionally, validating the MTRNet framework on diverse, cross-regional datasets will further refine the expert knowledge matrix and enhance its generalization capabilities across different rice cultivars globally. Future work should also include blinded expert evaluation of attribute-level outputs and near-OOD benchmarks containing weeds, other crops, and abiotic stress symptoms.

## 5. Conclusion

This study proposed MTRNet, a knowledge-guided multi-task framework for rice leaf disease diagnosis that combines expert-matrix-based auxiliary attribute supervision with a two-stage cascade for rejecting out-of-distribution inputs. On a public four-class dataset, we found that image-level splits are compromised by rotated and flipped near-duplicates; under source-aware cross-validation, MTRNet achieved an accuracy of 96.32% ± 1.45%, and the cascade rejected 77.78% ± 0.63% of far-OOD images at a false rejection rate of 8.29% ± 2.83%. The ablation showed that most rejections come from the color-based Stage I filter, while the knowledge-guided consistency check adds a small gain (about 1.5 percentage points) at a small false-rejection cost. Because attribute targets are derived from disease labels, the attribute heads provide structured, knowledge-guided outputs and regularization rather than evidence of independent feature disentanglement.

The work has several limitations. The evaluation relies on a single public dataset with limited source diversity and no acquisition metadata; the OOD set contains only far-OOD images (cats and dogs); and RGB images alone cannot reliably separate Tungro from nutrient deficiencies. Computationally, the ResNet-50 backbone (23.5 M parameters, 4.09 G MACs) limits deployment on low-power devices, and extending the expert matrix to new diseases requires manual rule definition and retraining. Future work will address these issues through external field validation, near-OOD benchmarks, lightweight and quantized backbones for edge inference, and scalable construction of attribute rules.

## Supporting information

S1 Appendix Near-duplicate analysis and supplementary evaluation details. Perceptual-hash near-duplicate analysis of the rice disease dataset with example image pairs; construction of the source-aware folds; per-fold results and pooled confusion matrix of the source-aware cross-validation; threshold-selection analysis on the OOD images; and a maximum-softmax-probability baseline for Stage II.
(DOCX)
