# Supplementary material for: Knowledge-guided multi-task learning with cascade out-of-distribution rejection for rice leaf disease diagnosis
Source: PLoS One. 2026 Nov 12;21(11):e0362080. doi: 10.1371/journal.pone.0362080 (PMC13759078; doi:10.1371/journal.pone.0362080)
Supplement: Supplementary file 1 [file pone.0362080.s001.docx]

**S1 Appendix. Near-duplicate analysis and supplementary evaluation details**

Supporting information for: Knowledge-Guided Multi-Task Learning with Cascade Out-of-Distribution Rejection for Rice Leaf Disease Diagnosis.

# 1. Near-duplicate analysis of the rice disease dataset

**Method.** Each of the 5,932 images was converted to grayscale, resized to 32 × 32 pixels, and transformed with a 2-D discrete cosine transform. The 8 × 8 lowest-frequency coefficients were binarized at their median to form a 64-bit perceptual hash (pHash). To make the comparison invariant to the rotations and flips used to enlarge the dataset, hashes were computed for all eight dihedral transformations (rotations by 0°, 90°, 180°, and 270°, each with and without horizontal flipping) and the minimum Hamming distance over these variants was used. Candidate matches were verified by the Pearson correlation between the query image and the correspondingly transformed matched image (128 × 128 grayscale).

**Table A. Nearest training-set neighbour of every validation and test image.**

| **Split (reference image-level split)** | **Images** | **Hamming = 0** | **Hamming ≤ 4** | **Hamming ≤ 8** | **Same class as match** | **Median pixel correlation** | **Pixel correlation ≥ 0.95** |
| --- | --- | --- | --- | --- | --- | --- | --- |
| Validation vs. training | 592 | 587 | 592 | 592 | 592 | 0.999 | 572 |
| Test vs. training | 595 | 585 | 595 | 595 | 595 | 0.999 | 569 |

*For comparison, the median pixel correlation between each test image and a randomly chosen training image was 0.001.*

**Table B. Near-duplicate clusters in the full dataset (union of all pairs within the threshold).**

| **Hamming threshold** | **Clusters** | **Mixed-class clusters** | **Clusters per class (Blast / Brown Spot / Blight / Tungro)** | **Median cluster size** | **Largest cluster** |
| --- | --- | --- | --- | --- | --- |
| ≤ 4 | 340 | 0 | 80 / 101 / 75 / 84 | 16 | 62 |
| ≤ 8 | 321 | 0 | 79 / 101 / 75 / 66 | 16 | 67 |
| ≤ 12 | 301 | 5 | 79 / 96 / 73 / 58 | 18 | 67 |

*The threshold of 8 bits was used for the source-aware folds because it yields class-pure clusters; at 12 bits, five clusters merge images of different classes, indicating hash collisions.*

**Fig A. Examples of test images (left of each pair) and their nearest training images (right of each pair).**

# 2. Source-aware cross-validation

**Fold construction.** Clusters obtained at a Hamming threshold of 8 were treated as source groups. Within each class, groups were shuffled (seed 42), sorted by size, and greedily assigned to the least-filled of ten bins, giving ten class-stratified bins of approximately equal size with no group split across bins. In fold k (k = 0,…,4), bins 2k and 2k+1 formed the test set (20%), bin (2k+2) mod 10 the validation set (10%), and the remaining seven bins the training set (70%). Training used the original protocol (ImageNet-initialized ResNet-50, Adam, learning rate 1×10⁻⁴, batch size 32, 50 epochs, loss weights 1.0/0.5/0.5/0.5, checkpoint with the highest validation accuracy) on an NVIDIA T4 GPU. The fold assignment is provided as source_groups_folds.csv in the code repository.

**Table C. Per-fold results of source-aware five-fold cross-validation (%).**

| **Fold** | **Train / Val / Test** | **Best val. acc.** | **Test acc.** | **Macro-F1** | **ORR Stage I** | **ORR VCC only** | **ORR full CIS** | **FRR Stage I** | **FRR VCC only** | **FRR full CIS** | **Attribute consistency** | **Training (min)** |
| --- | --- | --- | --- | --- | --- | --- | --- | --- | --- | --- | --- | --- |
| 0 | 4143 / 596 / 1193 | 98.66 | 97.82 | 97.83 | 76.3 | 10.7 | 78.3 | 11.82 | 0.42 | 12.24 | 99.58 | 43.2 |
| 1 | 4156 / 590 / 1186 | 93.90 | 94.35 | 94.65 | 76.3 | 7.4 | 77.5 | 7.17 | 0.51 | 7.67 | 99.49 | 43.6 |
| 2 | 4153 / 598 / 1181 | 96.82 | 96.44 | 96.43 | 76.3 | 5.4 | 77.2 | 4.49 | 0.93 | 5.42 | 99.07 | 43.6 |
| 3 | 4151 / 593 / 1188 | 99.49 | 97.56 | 97.62 | 76.3 | 6.4 | 78.6 | 9.76 | 0.51 | 10.02 | 99.49 | 43.5 |
| 4 | 4151 / 597 / 1184 | 100.00 | 95.44 | 95.59 | 76.3 | 6.9 | 77.3 | 5.07 | 1.01 | 6.08 | 98.99 | 43.6 |
| Mean ± SD |  | 97.77 ± 2.48 | 96.32 ± 1.45 | 96.43 ± 1.34 | 76.30 ± 0.00 | 7.36 ± 2.01 | 77.78 ± 0.63 | 7.66 ± 3.11 | 0.67 ± 0.28 | 8.29 ± 2.83 | 99.33 ± 0.28 | 43.5 |

*ORR is computed on the 1,000 OOD images; FRR and attribute consistency (fraction of test images with CS = 3) on the held-out positive images of each fold.*

**Table D. Pooled confusion matrix of the held-out predictions over the five folds (each of the 5,932 images predicted once).**

| **True \ Predicted** | **Leaf Blast** | **Brown Spot** | **Bacterial Leaf Blight** | **Tungro** | **Recall (%)** |
| --- | --- | --- | --- | --- | --- |
| Leaf Blast | 1340 | 37 | 63 | 0 | 93.06 |
| Brown Spot | 7 | 1578 | 15 | 0 | 98.62 |
| Bacterial Leaf Blight | 23 | 66 | 1495 | 0 | 94.38 |
| Tungro | 7 | 0 | 0 | 1301 | 99.46 |
| Precision (%) | 97.31 | 93.87 | 95.04 | 100.00 |  |

*Pooled accuracy 96.33%. Of the 218 misclassified images, 197 received attribute predictions fully consistent with the (wrong) predicted class.*

# 3. Separating threshold selection from evaluation

**Procedure.** The Stage I thresholds (S_min = 60, τ_env = 0.35) were originally selected with the same 1,000 OOD images that were used to report the ORR. To estimate the resulting optimism, the OOD images were randomly split 100 times into equal tuning and held-out halves. On each tuning half, (S_min, τ_env) were re-selected from S_min ∈ {20, 30, …, 100} and τ_env ∈ {0.05, 0.10, …, 0.65} by maximizing the OOD rejection rate subject to a Stage I retention on the validation images of the reference split of at least 94.09% (the retention of the original operating point). The full CIS, with Stage II predictions from the released reference checkpoint, was then evaluated on the held-out half.

**Table E. Threshold-selection analysis (100 random 50/50 splits of the OOD images).**

| **Quantity** | **Value** |
| --- | --- |
| Mean ORR on tuning halves | 81.22% |
| Mean ORR on held-out halves (± SD) | 81.04% ± 1.39% |
| 2.5th–97.5th percentile of held-out ORR | 78.59–83.91% |
| Mean optimism (tuning − held-out) | 0.18 percentage points |
| Held-out ORR with the original thresholds | 81.44% |
| Re-selected operating points (count / 100) | S_min = 60, τ_env = 0.35: 43; S_min = 50, τ_env = 0.4: 29; S_min = 40, τ_env = 0.45: 28 |

# 4. Maximum-softmax-probability baseline for Stage II

**Procedure.** To contextualize the contribution of the knowledge-guided consistency check, Stage II was replaced by a maximum-softmax-probability (MSP) threshold (ref. 31 in the main text), i.e., an input was rejected when the largest class probability fell below a threshold. For each fold, the threshold was set on the validation images so that the MSP rule rejected the same fraction of genuine validation images as the consistency check did, and the resulting cascade was evaluated on the held-out positive images and the 1,000 OOD images, using the saved predictions of the source-aware models (no retraining).

**Table F. Consistency check versus an MSP threshold as Stage II (mean ± SD over five folds).**

| **Configuration (source-aware CV)** | **ORR (%)** | **FRR (%)** |
| --- | --- | --- |
| Stage I + consistency check (MTRNet CIS) | 77.78 ± 0.63 | 8.29 ± 2.83 |
| Stage I + MSP (FRR-matched on validation) | 79.74 ± 3.72 | 9.21 ± 2.94 |
| MSP only | 16.82 ± 16.67 | — |

*Per-fold MSP thresholds: 0.488, 0.563, 0.538, 0.827, 0.957. The MSP-based cascade achieved a similar mean ORR but was markedly less stable across folds (per-fold ORR 76.8, 77.7, 76.7, 83.0, 84.5%) and had a slightly higher mean FRR.*

# 5. Computational cost

**Table G. Computational cost of MTRNet and the Cascade Inference System.**

| **Quantity** | **Value** |
| --- | --- |
| Parameters (total / auxiliary and diagnosis heads) | 23.53 M / 0.027 M |
| Multiply–accumulate operations, 224 × 224 input | 4.09 G (FLOPs 8.17 G) |
| Checkpoint size | 90 MB |
| Network latency, CPU (Intel Core i5-13500H), batch 1, 16 threads | 77.3 ± 5.7 ms |
| Network latency, CPU, batch 1, 1 thread | 172.0 ± 37.1 ms |
| Network latency, NVIDIA T4 GPU, batch 1 | 5.77 ms |
| Stage I (Bio-Grey Filter), CPU, per image | 0.34 ± 0.01 ms |
| Training time per fold (50 epochs, T4) | 43.5 min |

*Latency excludes image decoding. PyTorch 2.13 (CPU) and the Kaggle PyTorch build (GPU).*
